# Indoor Temperatures in Patient Waiting Rooms in Eight Rural Primary Health Care Centers in Northern South Africa and the Related Potential Risks to Human Health and Wellbeing

**DOI:** 10.3390/ijerph14010043

**Published:** 2017-01-06

**Authors:** Caradee Y. Wright, Renée A. Street, Nokulunga Cele, Zamantimande Kunene, Yusentha Balakrishna, Patricia N. Albers, Angela Mathee

**Affiliations:** 1 Environment and Health Research Unit, South African Medical Research Council, Pretoria 0001, South Africa; pnalbers@gmail.com; 2 Department of Geography, Geoinformatics and Meteorology, University of Pretoria, Pretoria 0002, South Africa; 3 Environment and Health Research Unit, South African Medical Research Council, Durban 4091, South Africa; renee.street@mrc.ac.za (R.A.S.); nokulunga.cele@mrc.ac.za (N.C.); 4 Discipline of Occupational and Environmental Health, University of KwaZulu-Natal, KwaZulu-Natal 4041, South Africa; 5 Environment and Health Research Unit, South African Medical Research Council, Doornfontein, Johannesburg 2094, South Africa; zama.kunene@mrc.ac.za (Z.K.); angie.mathee@mrc.ac.za (A.M.); 6 Biostatistics Unit, South African Medical Research Council, Durban 4091, South Africa; yusentha.balakrishna@mrc.ac.za; 7 University of Johannesburg, Doornfontein, Johannesburg 2094, South Africa; 8 University of the Witwatersrand, Braamfontein, Johannesburg 2000, South Africa

**Keywords:** indoor temperature, clinics, waiting rooms, rural, South Africa, climate change

## Abstract

Increased temperatures affect human health and vulnerable groups including infants, children, the elderly and people with pre-existing diseases. In the southern African region climate models predict increases in ambient temperature twice that of the global average temperature increase. Poor ventilation and lack of air conditioning in primary health care clinics, where duration of waiting time may be as long as several hours, pose a possible threat to patients seeking primary health care. Drawing on information measured by temperature loggers installed in eight clinics in Giyani, Limpopo Province of South Africa, we were able to determine indoor temperatures of waiting rooms in eight rural primary health care facilities. Mean monthly temperature measurements inside the clinics were warmer during the summer months of December, January and February, and cooler during the autumn months of March, April and May. The highest mean monthly temperature of 31.4 ± 2.7 °C was recorded in one clinic during February 2016. Maximum daily indoor clinic temperatures exceeded 38 °C in some clinics. Indoor temperatures were compared to ambient (outdoor) temperatures and the mean difference between the two showed clinic waiting room temperatures were higher by 2–4 °C on average. Apparent temperature (AT) incorporating relative humidity readings made in the clinics showed ‘realfeel’ temperatures were >4 °C higher than measured indoor temperature, suggesting a feeling of ‘stuffiness’ and discomfort may have been experienced in the waiting room areas. During typical clinic operational hours of 8h00 to 16h00, mean ATs fell into temperature ranges associated with heat–health impact warning categories of ‘caution’ and ‘extreme caution’.

## 1. Introduction

Exposure to high ambient temperature is associated with adverse human health effects [1,2,3]. Heat-related health effects can range from headaches and nausea to extreme events such as heat stroke and cardiac arrest [3]. Studies of both increased ambient temperature and mortality and the effect of heat waves have consistently shown that older age groups are more at risk [4,5]. Children and those with pre-existing diseases are also at particular risk from high temperature health effects. 

Climate predictions suggest ambient temperatures will increase. Model projections for temperature in a changing climate suggest elevated temperatures and more frequent heat waves, both of which would impact adversely on human health [6,7,8,9]. A recent study has shown that, due to climate change, the African continent is projected to see increases in the number of days when health may be adversely affected by elevated maximum apparent temperatures [10]. In South Africa, there is growing concern regarding extreme heat events and public health [9,11] in light of existing levels of poverty, inequality and other social determinants of health which may increase exposure of vulnerable groups to elevated temperatures, a climate-related health threat. 

In several countries around the world, including South Africa, a substantial portion of the country’s population relies on government-funded health care systems and infrastructure, including hospitals and clinics. Primary health care (PHC) centers or clinics vary in terms of resources and infrastructure. A commonly cited problem within the South African public health sector is that of duration of waiting times [12,13]. A study in a South African district hospital showed the average waiting time for stable patients (from arrival of the patient until the start of the consultation by the medical practitioner) was 545 min (~9 h) [13]. Sitting for hours in a clinic waiting room may pose health risks, particularly when the temperatures and relative humidity (RH) of such waiting rooms are elevated. A South African-based strategy, called ‘Ideal Clinic’, designed to respond to the current deficiencies in the quality of PHC services, has set the target for a maximum clinic waiting time at 3 h [14,15]. However, adjusting the waiting time for the clinic should also take into account other conditions such as the indoor clinic temperature.

The Limpopo Province is situated in the northernmost corner of South Africa, and incorporates both tropical and subtropical climates. An analysis of 21 years of temperature and rainfall data in the Limpopo Province suggested that maximum temperatures increased during the study period, indicating tendency towards local warming [16]. 

Mopani District in Limpopo Province is situated within the subtropical zone. During the summer months, maximum temperatures of 38 °C have been recorded [17]. Owing to increasing ambient temperatures and known long waiting periods in clinics without cooling systems, the aim of this study was to measure waiting room temperatures at rural primary health care centers over a five-month period in the southern hemisphere summer in the Mopani District Municipality, Limpopo Province.

## 2. Materials and Methods

### 2.1. Study Area and Sample

**Figure 1:**
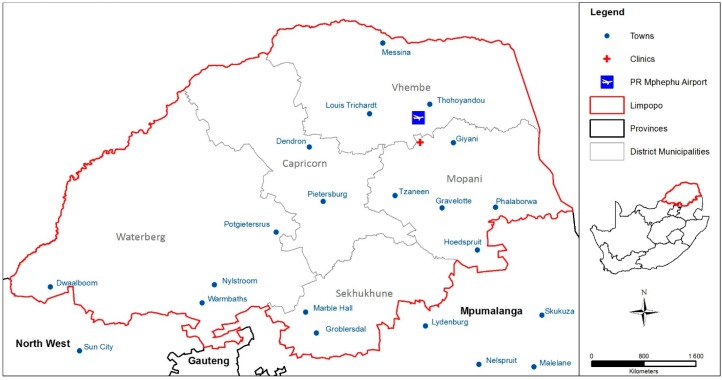
Map showing the location of Mopani District in Limpopo Province, South Africa (map produced in-house using geographic information system (GIS) software ArcGIS [18]).

The study was conducted in the Mopani District, Limpopo Province (Figure 1). The district has five sub-districts, Greater Tzaneen, Greater Giyani, Greater Letaba, Maruleng and Ba-Phalaborwa. We were provided with a list of possible clinic study sites from the Limpopo Provincial Department of Health. Five clinics were identified by the Public Health Manager of Mopani District in Greater Tzaneen, five in Greater Giyani, four in Greater Letaba, and three clinics in each of the Maruleng and Ba-Phalaborwa sub-districts. We aimed to install two instruments per clinic, therefore 40 loggers for the 20 clinics.

### 2.2. Procedures for Data Collection

The study was conducted in summer/autumn months from the 11 December 2015 to the 5 May 2016. Permission to install the temperature and RH loggers in the primary health care centers (clinics) was obtained from Limpopo Provincial Department of Health and the Mopani District Municipality. Research ethics clearance was granted from the South African Medical Research Council Research Ethics Committee (EC005-3/2014). All loggers were pre-tested and piloted in an office environment for a period of three days before the study to ensure the loggers were logging appropriately and consistently. Loggers were installed in clinics by two trained fieldworkers from the 11 to the 17 December 2015 (Table 1). 

The fieldworkers aimed to install loggers in each identified clinic using GPS coordinates for each clinic. However, challenges such as long distances between clinics, difficult field conditions and untarred roads made it difficult to reach all of the clinics in the allotted time. A total of 11 clinics were reached and two loggers were installed per clinic. Upon arrival at the clinic, permission was obtained from the Head Clinic Health Care Professional. A standardized logger installation sheet was used to note logger identity numbers, installation date, time, location of loggers in clinic and activation button pressed to start measurements. Similarly, upon collection of the loggers, the log sheets were used to note collection date and time. 

Loggers were installed in the main indoor patient waiting area of each clinic. They were installed in a location that was not visible to the general public, mounted vertically, at least 0.5 m below the ceiling, away from windows and radiant sources. Only the Head Clinic Health Care Professional knew the location of the loggers and she informed her staff so that the instruments remained in place for the duration of the study. The loggers were installed using tape and zip ties to prevent them becoming dislodged. Loggers were not installed on heat generating surfaces such as the tops of refrigerators or television sets (if present), in order to avoid recording of temperature generated by these appliances.

### 2.3. Temperature and Humidity Measurements

Automated LogTag HAXO-8 temperature and RH loggers (LogTag Recorders Limited, Northcote, Auckland, New Zealand) were used. The temperature and humidity readings were set to record at 30-min intervals and with battery life and data storage capability considered this provided for the 5 to 6-months measurement period. The logger has a real-time on board clock to ensure each recording is date-time stamped. The LogTag temperature range is −40 °C to +85 °C with sensitivity better than 0.1 °C and the humidity resolution is >0.1%.

### 2.4. Ambient Outdoor Temperature Data

For comparison purposes with the measured indoor temperatures only, ambient temperature and relative humidity data for the study days were retrieved from the Thohoyandou airport weather station (484 m above sea level) [19] and compared against the indoor temperature and RH readings at each clinic (~539 m above sea level). Neither airport nor clinics are within close proximity to major water bodies. The ambient data were provided in daily minimum, maximum and mean formats only. Study clinics were less than 50 km away from the Thohoyandou airport. 

### 2.5. Apparent Temperature Calculations

In addition to measured temperature, we also calculated apparent temperature (e.g., ‘realfeel’ conditions) using indoor temperature and RH measurements made in the clinics. Apparent temperature is an indicator of thermal sensation, can be used in indoor settings [20,21] and has been used before when considering the relationship between heat and health [22,23]. Apparent temperature was calculated using the 5-min temperature and RH measurements made by the data loggers and using Equation (1):

(1) *AT* = *Ta* + 0.33 × *e* − 0.70 × *ws* − 4.00

where *AT* is apparent temperature, *Ta* is measured dry bulb temperature (°C) in the primary health care centre, *e* is water vapour pressure (hPa) and *ws* is wind speed [24]. Given that this is an indoor setting, *ws* was set to 0. Water vapour pressure was calculated using the RH measurements made in the clinics and applying Equation (2) as follows:

(2) *e* = *rh*/100 × 6.105 × exp(17.27 × *Ta*/(237.7 + *Ta*))

where *rh* is relative humidity (%). The apparent temperature (*AT*) calculations for the study days were made for comparison and discussion purposes in relation to the measured indoor temperatures, and for comparison to the heat impacts described in Table 2. These threshold ranges were defined by the United States National Weather Service [25] and are applied since no African classification exists. Temperature ranges have been assigned particular effects on the body and labelled for caution, extreme, danger and extreme danger. Health effects include, for example in the caution (27–32 °C) range, fatigue possible with prolonged exposure and/or physical activity, and even heat stroke, as in the extreme danger classification (≥50 °C).

### 2.6. Data Processing and Statistical Analysis

The collected data were downloaded using the LogTag Analyzer software and then exported into Microsoft Excel version 16 (Microsoft, Tula, OK, USA) for quality control and data checking. Data were then exported into STATA version 14 (StataCorp, College Station, TX, USA) for further statistical analysis. Arithmetic means were calculated for temperature and RH using data taken from the two loggers installed within each clinic to provide one temperature and RH measurement per date and time-stamped 5 min interval for each clinic during the study period. Daily mean temperatures were calculated for each clinic and graphed against time to observe the change over the study period. A monthly mean, along with the standard deviation, 1st percentile and 99th percentile were also used to describe the data for each clinic. The monthly mean measured temperatures were compared to the ambient temperatures using the *t*-test. Pearson correlation coefficients (r) and 95% confidence intervals (CI) were calculated between the daily mean indoor and outdoor temperatures and the relationship was examined using linear regression. The monthly mean measured temperatures were compared to the ambient temperatures using the *t*-test. Results were considered statistically significant for *p* < 0.05.

## 3. Results

### 3.1. Sample Description

Ten sets of temperature loggers were retrieved from the eleven clinics where loggers were installed in December 2016. Data for analysis were available from eight of these clinics; data from two clinics were lost during the data download process, and one set of loggers were irretrievable from the clinic due to road access constraints and geographic location. Of the eight sets of clinic logger data available, three clinics were from Greater Giyani, three from Greater Letaba, one from Ba-Phalaborwa and one from Greater Tzaneen sub-district municipalities.

### 3.2. Indoor Temperature and Relative Humidity Measurements

**Figure 2:**
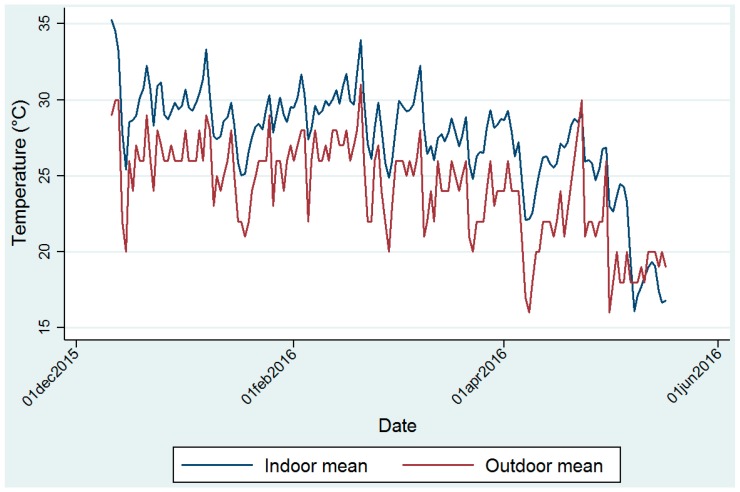
Daily mean indoorand outdoor temperatures.

Mean monthly temperature measurements inside the clinics were warmer during the summer months of December, January and February, and cooler during the autumn months of March, April and May (Figure 2). 

The highest mean monthly temperature of 31.4 ± 2.7 °C was recorded at clinic 3 in February 2016 (Table S1a). The lowest mean monthly temperature of 19.6 ± 3.7 °C was recorded in May 2016 Mean RH ranged from 43.2% ± 6.6% to 60.6% ± 8.8% (Table S1b). March 2016 was the month with the highest overall RH (55.5% ± 3.7%) from all combined clinics during the study period. Figure S1a,b illustrate the mean (over all time points) measured temperature per day for clinics 1 to 8 during the study period. Daily peaks are evident, exceeding 37 °C several times at clinics 1 and 3. A seasonal effect illustrates the southern hemisphere transition from summer to autumn (Figure 2) and the cooling effect of ambient temperature, thereby influencing indoor temperatures in the clinics. Mean temperature experienced at each time point during each month for clinic 1, as an illustration of daily variation in indoor temperatures measurements, shows that indoor temperature peaks in the mid-afternoon (Figure S2). Minimum indoor temperatures are typically measured before sunrise and before 6h00 in the morning. 

### 3.3. Ambient Temperature Data

Ambient (outdoor) mean (summarized in Figure 2), minimum and maximum temperature and RH measurements made at the Thohoyandou airport by month are given in detail in Table S2. The highest mean temperature was recorded in December 2015 at 26.2 ± 2.4 °C and the highest mean RH was recorded in March 2016 at 61.6% ± 15%.

### 3.4. Comparison between Indoor Measured Temperature and Ambient Outdoor Temperature

**Figure 3:**
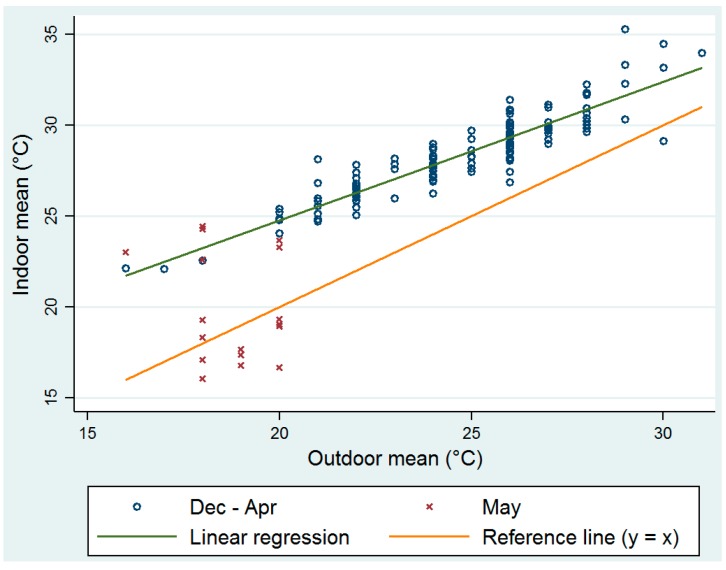
Linear regression for the period December to April between indoor and outdoor daily mean temperatures (intercept $\beta_{0}$ = 9.5, se($\beta_{0}$) = 0.72, slope $\beta_{1}$ = 0.8, se($\beta_{1}$) = 0.03, model R^2^ = 0.86).

There was strong correlation between indoor temperatures and the measured outdoor temperatures (Figure 3).

The correlation between the daily mean indoor and outdoor temperatures was 0.88 (CI: 0.83–0.91) and increased to 0.91 (CI: 0.88–0.94) when only the period December to April was considered. Monthly average temperatures were compared for each clinic to the ambient (outdoor) temperature measurements (Table S3). A large number of observations were available to calculate the mean monthly temperatures for the clinics (between 694 and 1488). This larger ‘sample size’ allowed for detection of smaller differences. 

One can see that the *p*-values are smallest (*p* < 0.0001) for the months December to April, and this is consistent with an almost 3 to 5 °C difference between the ambient and clinic temperatures. In May, we see relatively larger *p*-values and this is consistent with the smaller 1–2 °C difference between ambient and clinic temperatures. These results suggest that the period from December to April is when indoor temperatures are much higher than outdoor temperatures. Figure 3 shows the relationship between these two variables and it can be seen that temperatures for May are outliers. This suggests that the relationship changes from May. For this reason, the linear regression was fitted for the period December to April. Results show that indoor measured temperatures were significantly higher than outdoor temperatures (*p* < 0.001).

### 3.5. Calculated Clinic Indoor Apparent Temperature

**Figure 4:**
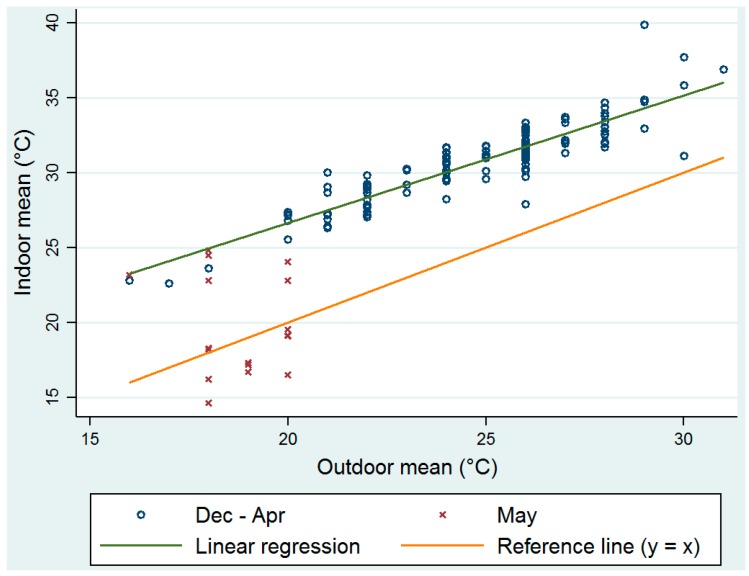
Linear regression for the period December to April between daily mean indoor apparent temperature and outdoor temperature (intercept $\beta_{0}$ = 9.6, se($\beta_{0}$) = 0.88, slope $\beta_{1}$ = 0.9, se($\beta_{1}$) = 0.04, model R^2^ = 0.811).

Mean AT per month for each clinic (Figure S3) with standard deviation and 1st and 99th percentiles (Table S3) shows that all measured clinic temperatures were statistically significantly higher than that of the daily maximum temperature measured at the weather station at Thohoyandou airport. Figure S3 illustrates the variation (between 1 and 6 °C) in difference between AT and ambient temperature per day for all clinics and over all time points. Figure 4 reflects the linear regression results between indoor apparent temperature and outdoor temperature and a similar relationship as found in Figure 3 can be seen here too.

### 3.6. Clinic Indoor Apparent Temperature and Potential Human Health Impacts during Patient Waiting Times

**Figure 5:**
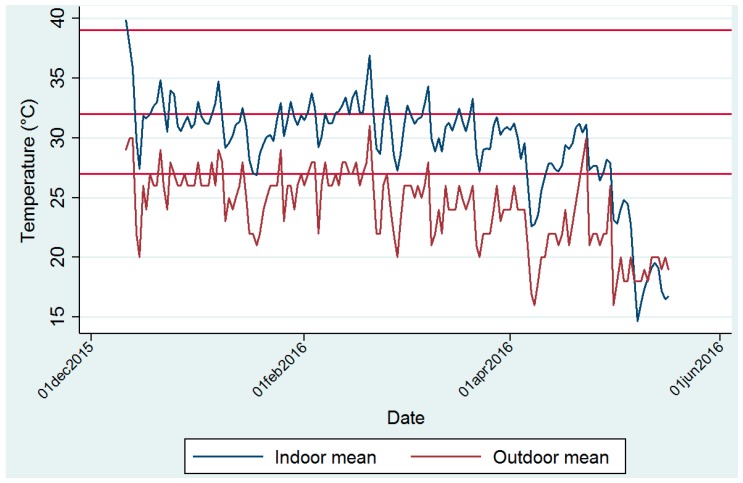
Daily mean indoor apparent temperature and outdoor temperature with superimposed threshold bands as described in Table 2.

Daily mean indoor AT and outdoor temperatures with superimposed health impact threshold bands are shown in Figure 5 (shown in Figure S4 by clinic). We superimposed red lines to reflect the temperature thresholds for health impacts as described in Table 2. One can see that during the study period, most of the days at all clinics fall within Bands I and II, namely the bands classified as caution and extreme caution. In Figure S5, we have focused on the period during the day when patients generally spend time waiting in clinics, i.e., between the working hours of 8h00 and 16h00. We compare the mean for all times of the day (blue) with those between 8h00 and 16h00 (red) but there was not much difference between the two. 

## 4. Discussion

One of the main ways in which climate change may affect health is an increase in extreme temperatures and an increase in the frequency and intensity of heat waves [26]. In assessing human health threats posed by climate change, it is vital to consider possible adaptation strategies [27]. Strategies such as meteorological early warning systems, timely public health and medical advice, scaling up of greening programs, urban planning and housing, and adequate health care and social support programs [28] can mitigate the detrimental health impacts from exposure to extreme temperatures. 

Primary health care clinics habitually rely on natural ventilation for infection control. Natural ventilation is the most practical measure and relies on the movement of air through open windows and doors, ensuring sufficient air changes per hour [29]. Our study has revealed the clinic temperatures were significantly higher than that of the ambient temperatures. The possible future risk of increasing outdoor temperature will influence clinic indoor temperatures, particularly in clinics without temperature control. A study describing 78 PHC clinics in KwaZulu-Natal (South Africa) revealed that 30 (59%) clinics had ceiling fans, and 18 (35%) had air conditioners that only provided temperature control [30]. In the National Core Standards for Health Establishments in South Africa [31] under facilities and infrastructure, one of the criteria is that ‘Appropriate ventilation is provided in theatres, patient accommodation and waiting areas’. Nonetheless ‘appropriate ventilation’ is not defined thereby leaving it up to interpretation. 

In clinic waiting rooms, individuals, some of whom may have underlying conditions which predispose them to infection, congregate in confined spaces and can be exposed to individuals who may be shedding potentially pathogenic microorganisms [32]. The situation may be compounded by increased temperatures considering that changes in infectious disease transmission patterns are a likely major consequence of climate change [33]. There are also thermal comfort considerations for clinic clients, as well as staff. Future studies should consider including information on window size, use of windows for ventilation and state of repair of windows in the clinics too. They may also consider the presence of mold and fungal growth which could proliferate on clinic walls and other surfaces in warm temperatures. A consequence of increasing thermal discomfort at clinics, especially in combination with protracted clinic waiting periods, may have detrimental effect on clinic attendance or health seeking behavior. 

The most effective short term adaptation measures for health include programs that implement or improve basic public health measures [34]. Our study findings support the need for controlled temperatures in clinic waiting rooms, as well as the reduction of clinic waiting times, provision of clean water for those waiting in clinics for protracted periods (especially older persons and young children), increased awareness amongst staff and clinic clients of the signs and symptoms of heat-related illness and ensuring that clinic properties are provided with trees or other forms of shade.

## 5. Conclusions

In the absence of indoor temperature control devices, clients waiting in clinics in settings such as the rural areas of the Limpopo province, and elsewhere around the world in developing countries with similar conditions, may be exposed to significantly higher temperatures indoors than outdoors. Health authorities may need to conduct clinic temperature assessments and implement client and staff-related strategies to reduce heat related hazards or ill health associated with clinic visits. 

## Figures and Tables

**Table 1:** Clinic location in sub-district and installation and collection date and time, as well as type of building and roof of each clinic.

Sub-District	Clinic No.	Installation		Collection		Type of Building and Roof
		Date	Time	Date	Time	
Greater Giyani	9	17 December 2015	- *	5 May 2016	- *	Brick walls, metal roof
	11	17 December 2015	- *	5 May 2016	- *	Brick walls, metal roof, wide front veranda
	3	11 December 2015	16:52	4 May 2016	9:17	Brick walls, metal roof
	1	11 December 2015	10:08	4 May 2016	10:31	Brick walls, metal roof
	8	11 December 2015	11:32	4 May 2016	12:32	Brick walls, metal roof
Greater Letaba	6	17 December 2015	9:40	5 May 2016	11:00	Brick walls, metal roof
	5	17 December 2015	12:00	4 May 2016	14:05	Brick walls, metal roof
	4	17 December 2015	13:20	5 May 2016	12:00	Brick walls, metal roof
	7	17 December 2015	10:55	5 May 2016	10:30	Brick walls, metal roof
Ba-Phalaborwa	2	11 December 2015	18:06	4 May 2016	10:00	Brick walls, metal roof
Greater Tzaneen	10	11 December 2015	- *	Not collected	- *	Brick walls, metal roof

* Missing information.

**Table 2:** Apparent temperature thresholds and potential health impacts at each threshold range [25].

Symptom Band	US NWS Classification	Apparent Temperature Range (°C)	US NWS Classified ‘Effect on Body‘
I	Caution	27–32	Fatigue possible with prolonged exposure and/or physical activity
II	Extreme caution	32–39	Heat stroke, heat cramps, or heat exhaustion possible with prolonged exposure and/or physical activity
III	Danger	39–51	Heat cramps or heat exhaustion likely, and heat stroke possible with prolonged exposure and/or physical activity
IV	Extreme danger	51	Heat stroke highly likely

US NWS: United States National Weather Service.

## Supplementary Materials

The following are available online at www.mdpi.com/1660-4601/14/1/43/s1, Figure S1: Indoor clinic temperatures, Figure S2: Mean indoor temperature experienced at each time point during each month for clinic 1, as an illustration of daily variation in indoor temperatures measurements, Figure S3: Indoor clinic apparent temperature, Figure S4: Differences between indoor clinic ambient apparent temperature and ambient temperature, Figure S5: Mean apparent temperature during clinic open hours of 8h00 to 16h00 compared to mean apparent temperature during all hours of the day, Table S1: Indoor clinic temperature and humidity measurements, Table S2: Ambient (outdoor) mean, minimum and maximum temperature and relative humidity measurements made at the Thohoyandou airport by month, Table S3: Monthly averages were compared for each clinic and the ambient (outdoor) temperature measurements and tested for statistically significant differences, Table S4: Mean apparent temperature (AT) per month for each clinic, with standard deviation and 1st and 99th percentiles.
