# Supplementary material for: Indoor Temperatures in Patient Waiting Rooms in Eight Rural Primary Health Care Centers in Northern South Africa and the Related Potential Risks to Human Health and Wellbeing
Source: Int J Environ Res Public Health. 2017 Jan 6;14(1):43. doi: 10.3390/ijerph14010043 (PMC5295294; doi:10.3390/ijerph14010043)
Supplement: Supplementary file 1 [file ijerph-14-00043-s001.pdf]

Supplementary Materials: Indoor Temperatures in Patient Waiting Rooms in Eight Rural Primary Health Care Centers in Northern South Africa and the Related Potential Risks to Human Health and Wellbeing

Caradee Y. Wright, Renee A. Street, Nokulunga Cele, Zamantimande Kunene, Yusentha Balakrishna, Patricia Albers and Angela Mathee

Table S1. Indoor clinic temperature and humidity measurements. (**a**) Mean measured temperature per month for each clinic (1–8) with standard deviation and 1st and 99th percentiles; (**b**) Mean relative humidity per month for each clinic with standard deviation and 1st and 99th percentiles.

(**a**)

	**Dec 2015**			**Jan 2016**			**Feb 2016**			**Mar 2016**			**Apr 2016**			**May 2016**		
	**Mean (SD)**	**p1**	**p99**	**Mean (SD)**	**p1**	**p99**	**Mean (SD)**	**p1**	**p99**	**Mean (SD)**	**p1**	**p99**	**Mean (SD)**	**p1**	**p99**	**Mean (SD)**	**p1**	**p99**
**1**	31.2 (2.9)	24.9	37.4	29.9 (2.5)	24.5	36.9	30.6 (2.8)	25.2	38.0	29.4 (2.4)	24.6	35.6	27.5 (2.9)	21.7	33.6	20.1 (4.0)	9.4	29.2
**2**	28.1 (2.3)	23.9	34.1	27.3 (2.0)	23.5	32.6	28.1 (2.1)	24.4	34.0	27.4 (2.0)	23.5	33.6	25.7 (2.2)	21.6	31.3	20.1 (4.8)	9.4	35.0
**3**	31.9 (2.9)	25.3	38.5	30.8 (2.4)	25.4	37.6	31.4 (2.7)	26.0	38.7	30.1 (2.4)	25.6	37.1	28.3 (2.6)	22.9	34.1	20.3 (4.2)	9.4	29.6
**4**	27.8 (2.1)	24.2	33.9	27.1 (2.3)	22.6	33.9	27.6 (2.5)	22.8	34.5	26.3 (2.2)	22.2	33.0	24.7 (2.1)	20.7	29.8	19.7 (4.5)	8.9	35.6
**5**	29.1 (1.8)	25.5	33.9	27.8 (2.3)	23.3	35.0	28.1 (2.5)	22.2	33.9	27.2 (2.0)	23.4	32.8	25.6 (2.2)	21.4	30.8	19.9 (4.8)	9.2	34.4
**6**	29.7 (2.1)	24.2	33.9	28.4 (2.4)	23.1	35.8	29.1 (2.7)	24.0	36.9	27.6 (2.3)	23.0	34.4	25.7 (2.5)	21.0	31.5	19.6 (3.7)	9.3	28.8
**7**	29.4 (2.4)	24.8	36.0	28.8 (2.5)	23.8	36.2	28.8 (2.6)	24.2	35.2	27.1 (2.3)	22.6	32.9	24.9 (2.8)	19.7	31.1	19.7 (4.5)	9.1	34.4
**8**	31.2 (2.9)	24.9	37.4	29.9 (2.5)	24.5	36.9	30.6 (2.8)	25.2	38.0	29.4 (2.4)	24.6	35.6	27.5 (2.9)	21.7	33.6	20.1 (4.0)	9.4	29.2
**Total**	29.8 (1.5)	27.8	31.9	28.8 (1.3)	27.1	30.8	29.3 (1.4)	27.6	31.4	28.0 (1.4)	26.3	30.1	26.2 (1.3)	24.7	28.3	19.9 (0.3)	19.6	20.3

(**b**)

	**Dec 2015**			**Jan 2016**			**Feb 2016**			**Mar 2016**			**Apr 2016**			**May 2016**		
	**Mean (SD)**	**p1**	**p99**	**Mean (SD)**	**p1**	**p99**	**Mean (SD)**	**p1**	**p99**	**Mean (SD)**	**p1**	**p99**	**Mean (SD)**	**p1**	**p99**	**Mean (SD)**	**p1**	**p99**
**1**	43.8 (6.1)	35.6	58.5	44.5 (5.9)	29.2	56.6	45.2 (6.8)	35.1	60.3	51.1 (7.8)	35.0	63.5	46.5 (4.0)	38.1	54.8	49.0 (7.9)	34.4	61.3
**2**	51.4 (4.9)	41.1	61.9	53.2 (5.6)	36.7	64.9	52.6 (6.5)	42.3	65.6	58.7 (7.7)	41.0	73.3	55.6 (3.7)	46.4	61.9	49.6 (10.2)	24.0	63.6
**3**	43.6 (5.5)	34.5	56.8	44.5 (5.4)	29.5	56.1	44.5 (6.6)	34.5	58.9	50.8 (7.4)	34.6	64.4	48.4 (3.5)	40.2	55.0	49.6 (8.2)	34.5	62.5
**4**	50.2 (3.8)	41.9	59.8	51.8 (6.2)	34.1	62.9	53.4 (6.3)	41.7	66.0	60.6 (8.3)	41.3	72.8	56.7 (3.0)	49.2	62.4	51.3 (9.9)	23.9	65.0
**5**	45.9 (4.6)	37.0	57.8	48.4 (6.8)	29.6	60.5	50.1 (7.3)	38.1	65.8	56.7 (8.4)	38.2	69.6	51.3 (3.8)	42.4	58.0	48.3 (10.1)	23.2	62.9
**6**	45.4 (6.3)	33.7	60.2	48.7 (7.8)	27.2	60.4	49.4 (8.2)	32.6	64.6	56.2 (8.9)	36.4	68.3	52.2 (4.3)	42.1	59.4	50.6 (7.9)	33.6	63.0
**7**	43.2 (6.6)	31.6	59.5	45.3 (7.2)	25.7	57.9	47.8 (8.2)	31.5	64.4	54.5 (10.2)	33.2	69.9	51.3 (6.2)	36.5	62.3	49.6 (9.9)	24.8	64.0
**8**	43.8 (6.1)	35.6	58.5	44.5 (5.9)	29.2	56.6	45.2 (6.8)	35.1	60.3	51.1 (7.8)	35.0	63.5	46.5 (4.0)	38.1	54.8	49.0 (7.9)	34.4	61.3
**Total**	45.9 (3.2)	43.2	51.4	47.6 (3.5)	44.5	53.2	48.5 (3.4)	44.5	53.4	55.0 (3.7)	50.8	60.6	51.1 (3.8)	46.5	56.7	49.6 (0.9)	48.3	51.3

SD: standard deviation; p1: 1st percentile; p99: 99th percentile.

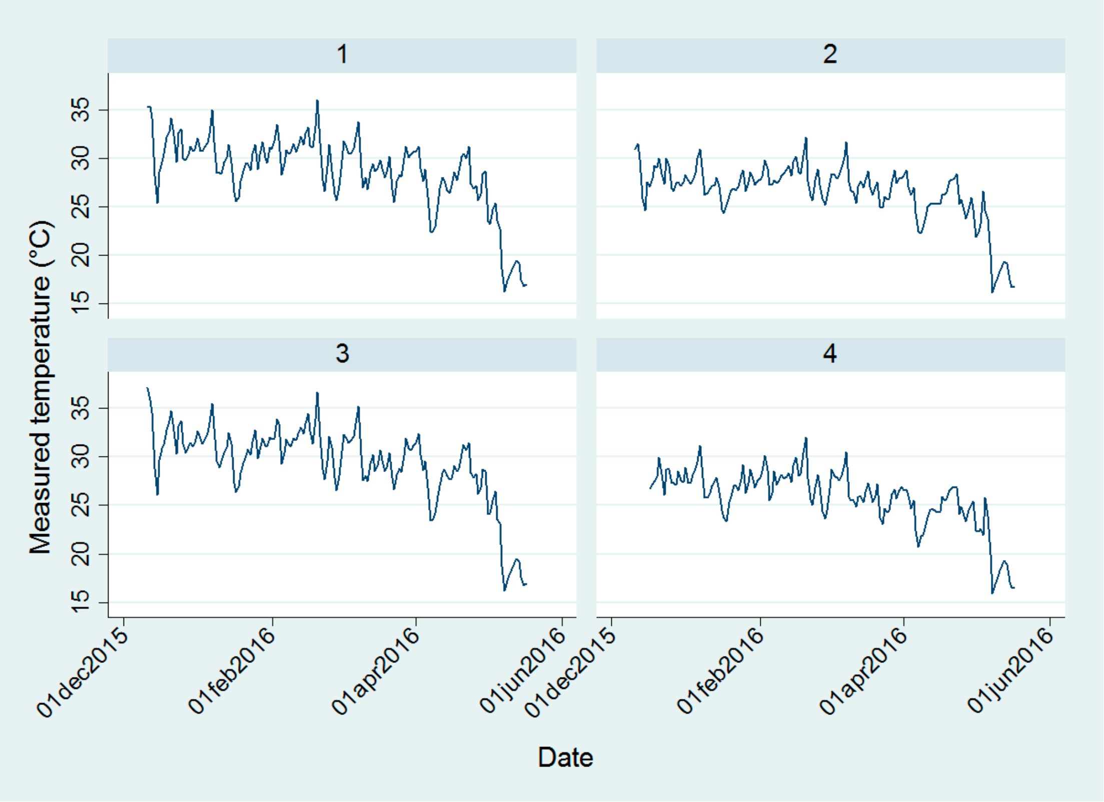

(**a**)

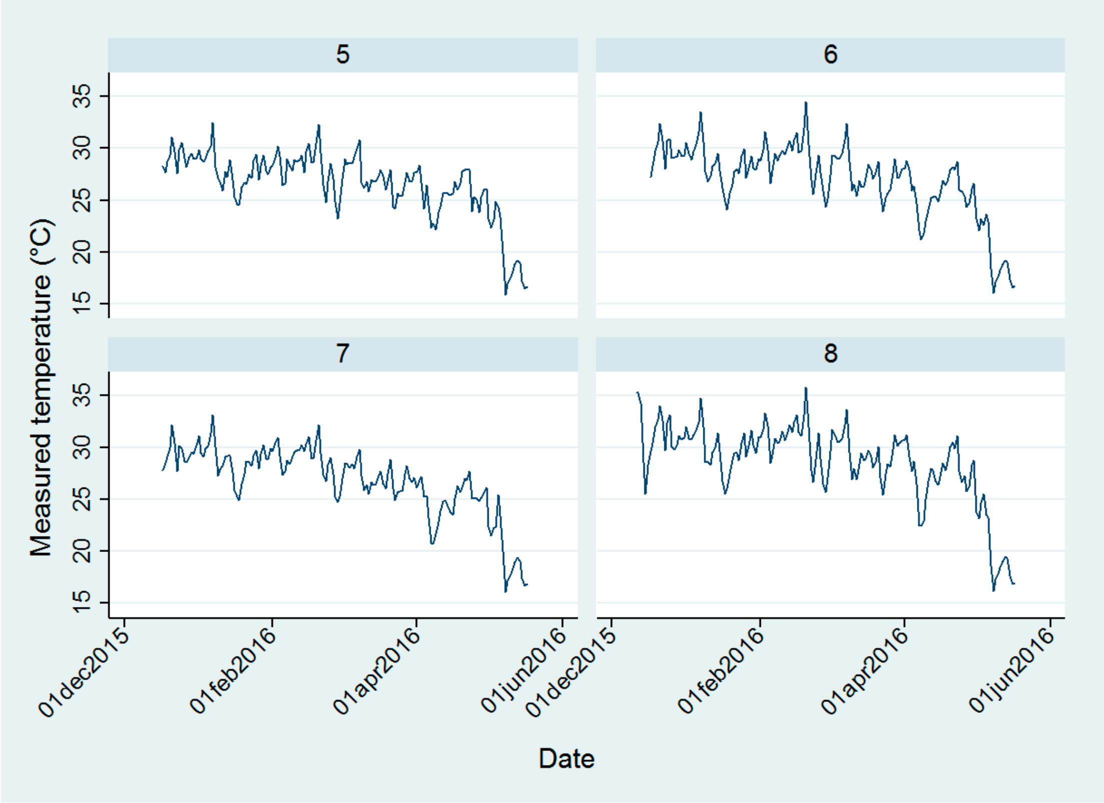

(**b**)

Figure S1. Indoor clinic temperatures. (**a**) Mean (over all time points) measured indoor temperature per day for clinics 1–4; (**b**) Mean (over all time points) measured indoor temperature per day for clinics 5–8.

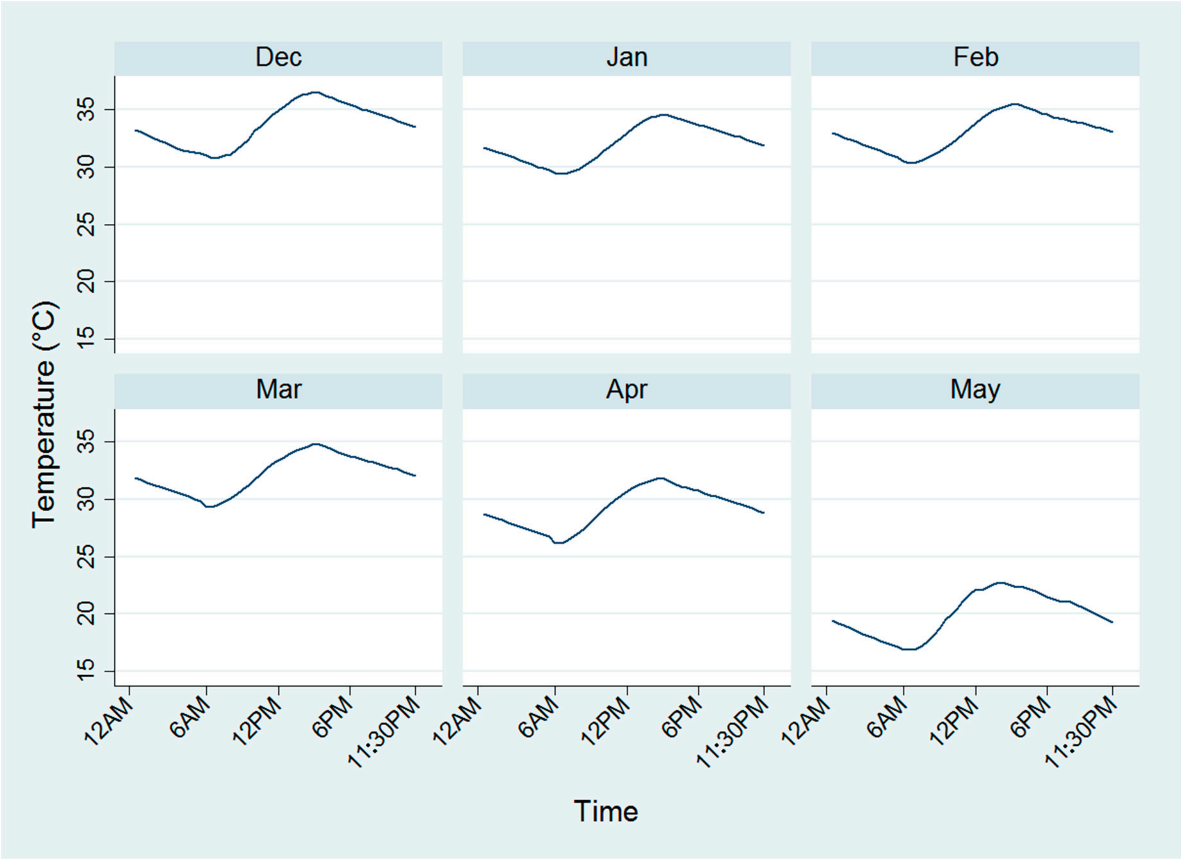

Figure S2. Mean indoor temperature experienced at each time point during each month for clinic 1, as an illustration of daily variation in indoor temperatures measurements.

Table S2. Ambient (outdoor) mean, minimum and maximum temperature and relative humidity measurements made at the Thohoyandou airport by month.

	Dec 2015			Jan 2016			Feb 2016			Mar 2016			Apr 2016			May 2016		
	**Mean (SD)**	**p1**	**p99**	**Mean (SD)**	**p1**	**p99**	**Mean (SD)**	**p1**	**p99**	**Mean (SD)**	**p1**	**p99**	**Mean (SD)**	**p1**	**p99**	**Mean (SD)**	**p1**	**p99**
**Min temp**	20.4 (1.4)	18.0	23.0	20.1 (1.9)	15.0	24.0	20.6 (1.3)	18.0	22.0	19.2 (2.1)	15.0	22.0	16.2 (2.4)	13.0	22.0	12.4 (2.1)	10.0	16.0
**Max temp**	31.9 (4.1)	21.0	38.0	30.7 (3.4)	24.0	39.0	31.4 (4.5)	22.0	40.0	29.2 (3.7)	22.0	36.0	27.5 (4.6)	18.0	35.0	24.8 (2.9)	18.0	29.0
**Mean temp**	26.2 (2.4)	20.0	30.0	25.4 (2.1)	21.0	29.0	25.9 (2.5)	20.0	31.0	24.2 (1.9)	20.0	28.0	22.1 (2.9)	16.0	30.0	18.8 (1.1)	16.0	20.0
**Min humidity**	28.0 (17.2)	13.0	78.0	28.1 (13.2)	7.0	62.0	31.7 (20.5)	9.0	80.0	38.5 (19.0)	11.0	72.0	34.7 (15.7)	12.0	67.0	29.8 (10.6)	17.0	50.0
**Max humidity**	70.4 (11.5)	57.0	94.0	69.9 (12.8)	41.0	88.0	68.7 (15.1)	26.0	88.0	79.5 (9.2)	59.0	94.0	77.5 (9.6)	45.0	90.0	77.5 (6.6)	62.0	85.0
**Mean humidity**	48.9 (13.7)	32.0	83.0	48.9 (13.4)	24.0	76.0	50.7 (16.8)	26.0	85.0	61.1 (15.0)	33.0	85.0	57.4 (11.7)	30.0	75.0	54.8 (7.1)	42.0	67.0

Table S3. Monthly averages were compared for each clinic and the ambient (outdoor) temperature measurements and tested for statistically significant differences.

**Month**	**Dec 2015**		**Jan 2016**		**Feb 2016**		**Mar 2016**		**Apr 2016**		**May 2016**	
**Ambient Temperature °C (SD)**	**26.2 (2.4)**		**25.4 (2.1)**		**25.9 (2.5)**		**24.2 (1.9)**		**22.1 (2.9)**		**18.8 (1.1)**	
**Clinic**	**Temp**	***p*-Value**	**Temp**	***p*-Value**	**Temp**	***p*-Value**	**Temp**	***p*-Value**	**Temp**	***p*-Value**	**Temp**	***p*-Value**
**1**	31.2 (2.9)	<0.0001	29.9 (2.5)	<0.0001	30.6 (2.8)	<0.0001	29.4 (2.4)	<0.0001	27.5 (2.9)	<0.0001	20.1 (4.0)	0.0002
**2**	28.1 (2.3)	0.002	27.3 (2.0)	<0.0001	28.1 (2.1)	0.0001	27.4 (2.0)	<0.0001	25.7 (2.2)	<0.0001	20.1 (4.8)	0.0003
**3**	31.9 (2.9)	<0.0001	30.8 (2.4)	<0.0001	31.4 (2.7)	<0.0001	30.1 (2.4)	<0.0001	28.3 (2.6)	<0.0001	20.3 (4.2)	<0.0001
**4**	27.8 (2.1)	0.007	27.1 (2.3)	0.0001	27.6 (2.5)	0.001	26.3 (2.2)	<0.0001	24.7 (2.1)	0.0001	19.7 (4.5)	0.007
**5**	29.1 (1.8)	<0.0001	27.8 (2.3)	<0.0001	28.1 (2.5)	0.0001	27.2 (2.0)	<0.0001	25.6 (2.2)	<0.0001	19.9 (4.8)	0.002
**6**	29.7 (2.1)	<0.0001	28.4 (2.4)	<0.0001	29.1 (2.7)	<0.0001	27.6 (2.3)	<0.0001	25.7 (2.5)	<0.0001	19.6 (3.7)	0.013
**7**	29.4 (2.4)	<0.0001	28.8 (2.5)	<0.0001	28.8 (2.6)	<0.0001	27.1 (2.3)	<0.0001	24.9 (2.8)	<0.0001	19.7 (4.5)	0.007
**8**	31.2 (2.9)	<0.0001	29.9 (2.5)	<0.0001	30.6 (2.8)	<0.0001	29.4 (2.4)	<0.0001	27.5 (2.9)	<0.0001	20.1 (4.0)	0.0002

Table S4. Mean apparent temperature (AT) per month for each clinic, with standard deviation and 1st and 99th percentiles.

	Dec 2015			Jan 2016			Feb 2016			Mar 2016			Apr 2016			May 2016		
	**Mean (SD)**	**p1**	**p99**	**Mean (SD)**	**p1**	**p99**	**Mean (SD)**	**p1**	**p99**	**Mean (SD)**	**p1**	**p99**	**Mean (SD)**	**p1**	**p99**	**Mean (SD)**	**p1**	**p99**
**1**	33.7 (3.5)	26.4	41.6	32.0 (2.8)	26.0	39.0	33.0 (3.2)	27.4	41.8	32.2 (2.7)	26.6	38.5	29.2 (3.5)	21.9	36.9	19.9 (4.6)	6.9	30.5
**2**	30.5 (2.8)	25.6	38.3	29.7 (2.3)	25.5	35.4	30.6 (2.6)	26.4	38.1	30.5 (2.4)	25.7	36.8	27.7 (2.9)	22.1	34.6	19.9 (5.4)	6.7	35.6
**3**	34.6 (3.5)	26.9	42.6	33.3 (2.7)	27.2	39.8	34.2 (3.1)	28.3	42.7	33.1 (2.7)	27.7	40.2	30.4 (3.3)	23.4	37.5	20.3 (4.9)	6.8	30.9
**4**	30.0 (2.7)	25.3	38.1	29.2 (2.7)	24.0	36.5	30.1 (3.0)	24.3	38.8	29.1 (2.6)	24.3	36.5	26.5 (2.8)	21.3	33.2	19.6 (5.1)	6.2	36.0
**5**	31.2 (2.2)	26.4	37.2	29.8 (2.5)	24.3	36.5	30.3 (2.8)	24.0	37.5	29.8 (2.3)	25.4	35.9	27.2 (2.8)	21.8	33.6	19.6 (5.3)	6.5	35.0
**6**	31.9 (2.3)	28.0	38.1	30.5 (2.5)	24.3	37.0	31.5 (2.9)	26.2	39.7	30.4 (2.5)	24.7	36.8	27.4 (3.0)	21.6	34.1	19.5 (4.3)	6.7	29.7
**7**	31.2 (2.7)	26.1	38.5	30.6 (2.6)	24.9	37.7	31.0 (2.7)	26.2	38.0	29.5 (2.7)	23.8	35.7	26.2 (3.4)	20.0	33.0	19.5 (5.0)	6.4	35.3
**8**	33.7 (3.6)	26.4	41.6	32.0 (2.8)	26.0	39.0	33.0 (3.2)	27.4	41.9	32.2 (2.7)	26.6	38.5	29.2 (3.5)	21.9	36.9	20.0 (4.7)	6.9	30.5

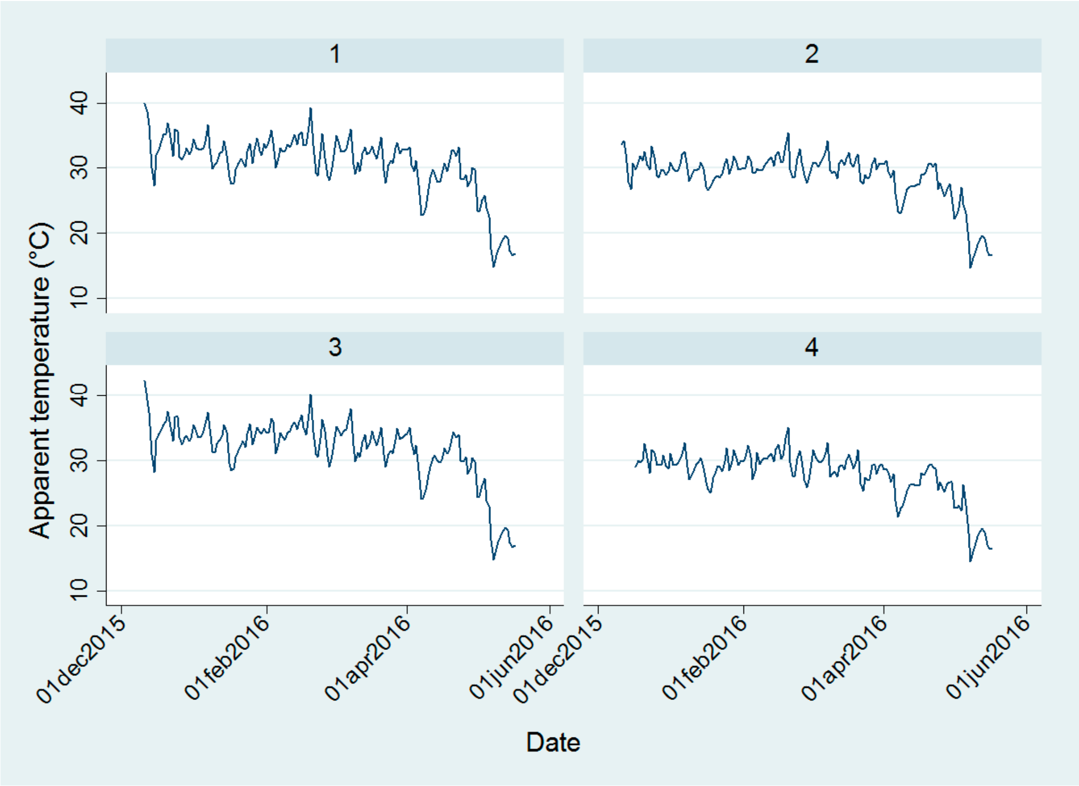

(**a**)

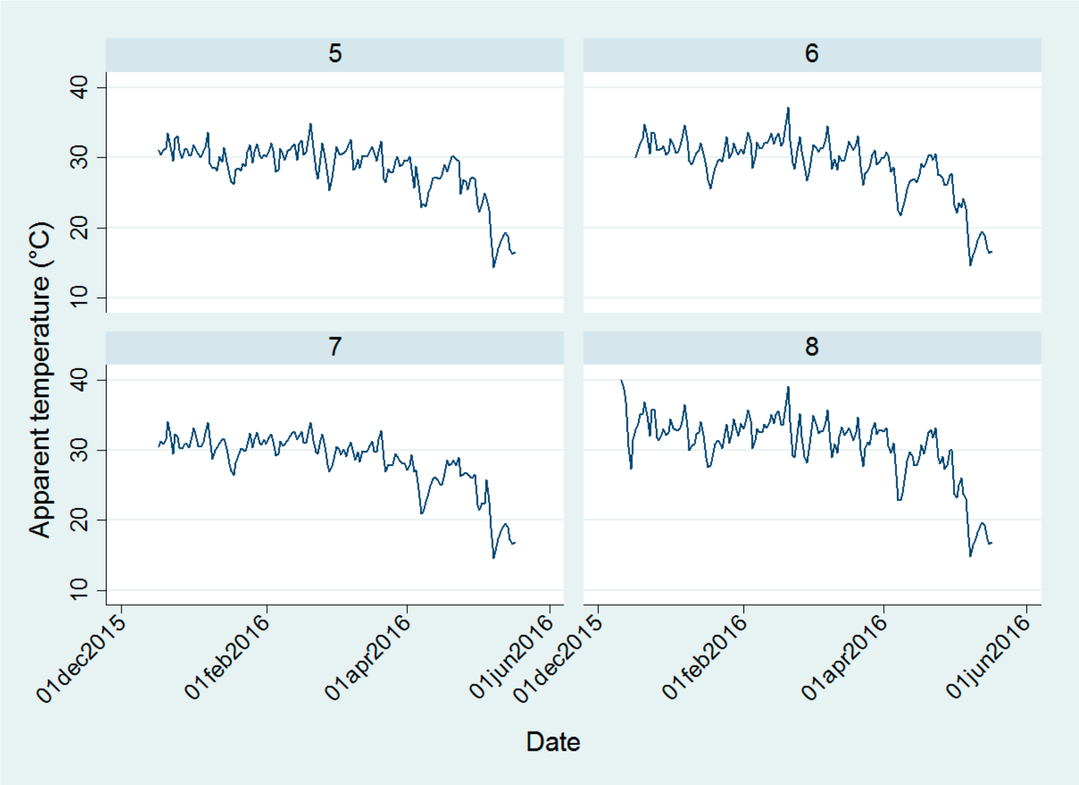

(**b**)

Figure S3. Indoor clinic apparent temperature. (**a**) Mean (over all time points) AT per day for clinics 1–4; (**b**) Mean (over all time points) AT per day for clinics 5–8.

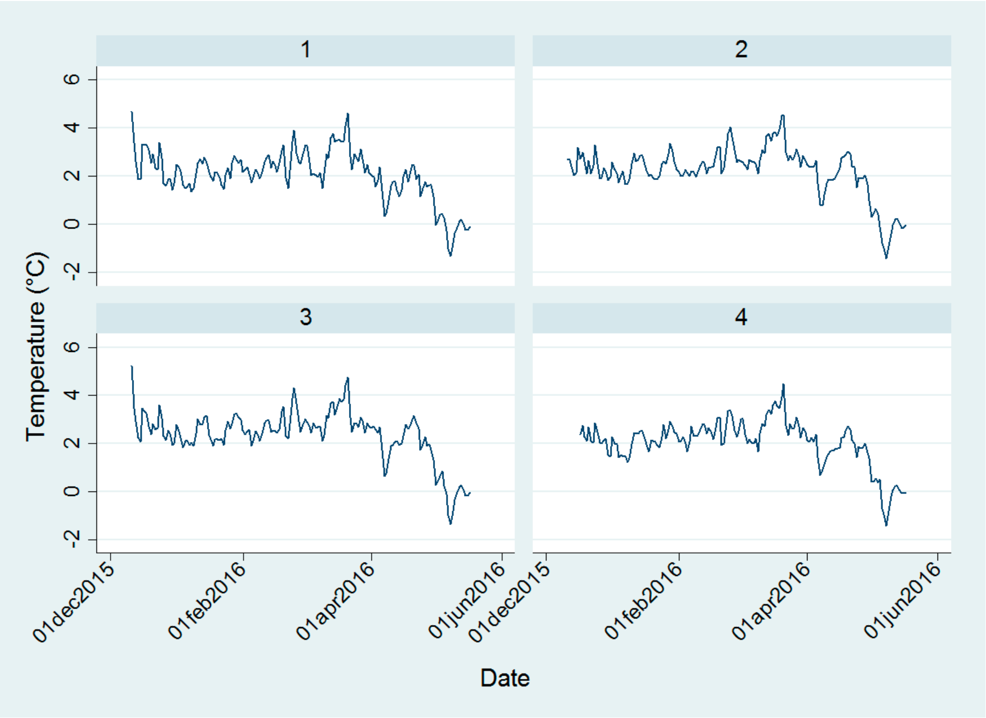

(**a**)

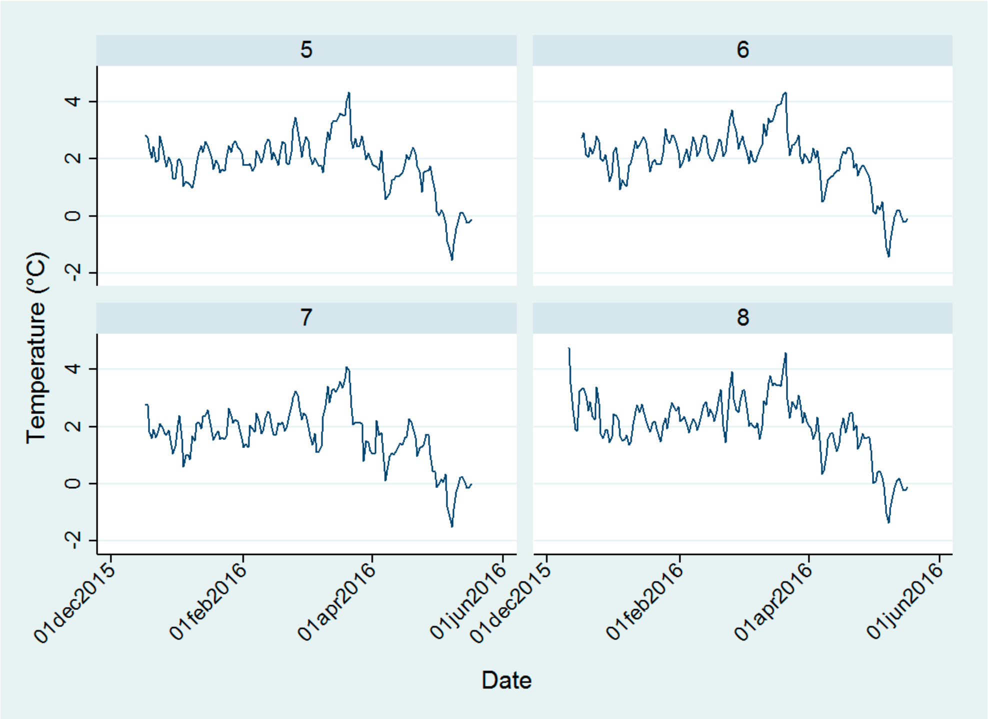

(**b**)

Figure S4. Differences between indoor clinic ambient apparent temperature and ambient temperature. (**a**) Mean (over all time points) difference between AT and ambient temperature per day for clinics 1–4; (**b**) Mean (over all time points) difference between AT and ambient temperature per day for clinics 5–8.

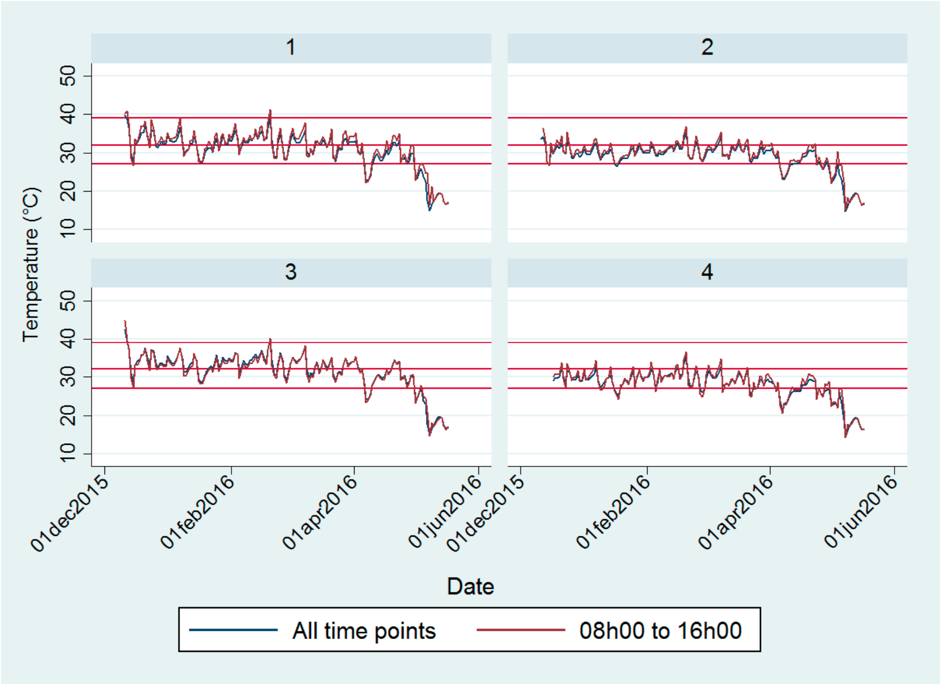

(**a**)

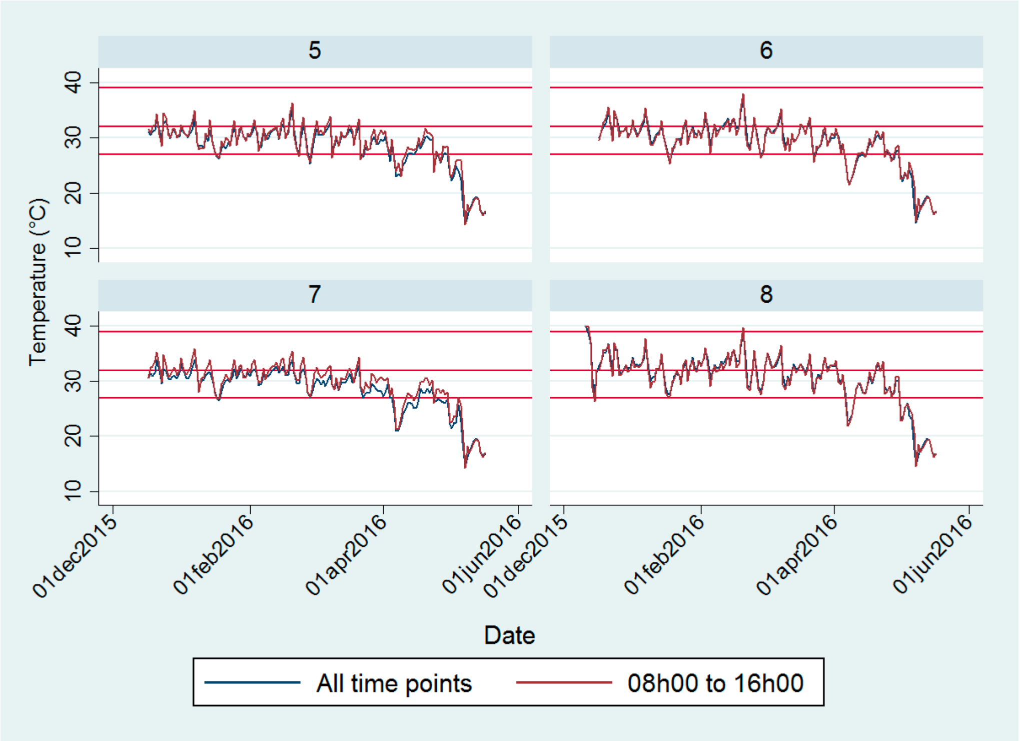

(**b**)

Figure S5. Mean apparent temperature during clinic open hours of 8h00 to 16h00 compared to mean apparent temperature during all hours of the day. (**a**) Mean (for the period 8h00 to 16h00) AT (red) and mean (over all time points) (blue) AT per day for clinics 1–4; (**b**) Mean (for the period 8h00 to 16h00) AT (red) and mean (over all time points) (blue) AT per day for clinics 5–8. Red lines on the plots depict symptom bands from Table 2 where symptom band I between 32–39 °C is Caution (bottom and middle lines) and symptom band II between 32–39 °C is Extreme caution (middle and top lines).
